# Inversion-free image recovery from strong aberration using a minimally sampled transmission matrix

**DOI:** 10.1038/s41598-018-38027-y

**Published:** 2019-02-04

**Authors:** Kwanjun Park, Taeseok Daniel Yang, Hyung-Jin Kim, Taedong Kong, Jung Min Lee, Hyuk Soon Choi, Hoon Jai Chun, Beop-Min Kim, Youngwoon Choi

**Affiliations:** 1 Department of Bio-convergence Engineering, Korea University, Seoul, 02841 South Korea; 2 School of Biomedical Engineering, Korea University, Seoul, 02841 South Korea; 3 Department of Gastroenterology and Hepatology, Korea University, Seoul, 02841 South Korea

## Abstract

A transmission matrix (TM), a characteristic response for an input-output relation of an optical system, has been used for achieving diffraction-limited and aberration-free images through highly-aberrant imaging systems. However, its requirement of acquiring a huge-size TM along with its heavy computational load limit its widespread applications. Here we propose a method for TM-based image reconstruction, which is more efficient in terms of data manipulation and computational time. Only 10% of the TM elements for a fish-eye (FE) lens with strong aberration were sampled compared to that required for the image reconstruction by the conventional inversion method. The missing information was filled in by an iterative interpolation algorithm working in *k*-space. In addition, as a replacement of the time-consuming matrix inversion process, a phase pattern was created from the minimally sampled TM in order to compensate for the angle-dependent phase retardation caused by the FE lens. The focal distortion could be corrected by applying the phase correction pattern to the angular spectrums of the measured object images. The remaining spatial distortion could also be determined through the geometrical transformation also determined by the minimally sampled TM elements. Through the use of these procedures, the object image can be reconstructed 55 times faster than through the use of the usual inversion method using the full-sized TM, without compromising the reconstruction performances.

## Introduction

Accurate visualization of an object of interest often provides immediate solutions for problems under consideration. Optical imaging has played a crucial role in object visualization under the investigation in wide-ranging areas due to its good performance, such as high-resolution, high-speed, non-toxicity, low-cost, system compactness, etc. These days, the demand is on the rise for compact imaging devices that serve superior imaging performances but are smaller in size. For instance, the requirements for the cameras equipped in mobile phones, wireless medical endoscopes, portable military night vision devices, pilotless drones, etc. necessitate small size and light weight for the respective constraints of space and weight. For this compact equipment, using the optical elements in small sizes without losing any performance is essential in not compromising the imaging quality.

These optics with small dimensions often have large surface curvatures in non-spherical shapes so as to cover wide fields of view through the limited apertures. However, due to the requirement for the simple optical configuration, the imaging systems with these lenses show inevitable aberration and distortion over the field of view. This aberration causes the formation of a deformed focal plane which does not fit on a flat detector chip, resulting in significant regional blurring on the taken images due to the partial defocus. In addition, the variation of magnification along the radial direction causes image deformation, which is known to appear as a pincushion or a barrel distortion. These two types of distortion, focal and spatial distortion, make the compact optical systems equipped with wide-viewing lenses in such small dimensions non-suitable for high-resolution and high-precision measurements.

Extensive efforts have been made to correction of the aberration and distortion caused by either optical systems or imaging environments. The iterative wavefront control techniques based on the adaptive optics (AO) have played a key role for this purpose. Astronomers have corrected the aberration and distortion induced by the air turbulence using deformable mirrors^1–3^. In microscopy, improvement of axial and lateral resolution, as well as enhancement of image quality of laser scanning microscopy for human eyes^4,5^, two-photon fluorescence or reflectance microscopy for biological tissues^6–8^, multi-harmonic generation microscopy^9,10^, and confocal microscopy^11–13^ were achieved using the adaptive optics technique in conjunction with the wavefront sensing method. Recently, the environmental aberration was shown to be corrected with the AO technique in the presence of multiple scattering^14^. These methods have been successful in suppressing the image deterioration induced by the aberrant effect, but the level of complexity required for their implementation is very high due to the requirement for the wavefront control by optical instruments such as spatial light modulators (SLMs), digital micro-mirror devices (DMDs), and deformable mirrors (DMs).

In order to bypass the sophisticated optical alignment associated with the light controlling devices, the post image processing method based on the information of the system’s optical response has been intensively exploited. Measuring a transmission matrix (TM) is one of the methods for describing the systems’ optical responses in the most informative way. TMs have proven to be useful for the correction of the aberration occurring in optical imaging systems. Usually, the input-output responses of the optical systems of interest are measured in a form of a matrix characterizing their complete transmission properties^15–21^. This approach is often used for the removal of multiple scattering effects in disordered media such as biological tissues. The wave modification induced by optical systems during light transmission can be considered as a simple transformation for the wavefront, which is well described by the system’s TM. Thus, the correction of the induced distortion is performed by applying the inverse of the measured TM to the taken images regardless of whether the wavefront modification is caused by scattering or aberration. Despite the mathematical simplicity and the superior correction accuracy, however, this method is highly limited in a wide range of applications due to its need for huge data sets associated with TMs. Since the number of TM elements quadratically depends on the recovered numerical aperture (NA), attaining numerous TM elements is the strict requirement for high-resolution image reconstruction^22,23^. This causes first difficulties in acquiring and handling the big-size data, then results in the time-consuming data processing associated with the inversion of the TM. Thus, the TM-based image reconstruction has lagged far behind real-time imaging, even in the case of simple aberration correction.

In this article, we present a method for aberration correction for images taken through a highly aberrant optical system equipped with a fish-eye (FE) lens detached from a capsule-type wireless endoscope. Our method is based on the TM measurement, but works with a minimally sampled set of TM elements of which size is 10% of the full-sized TM. In addition, unlike the usual reconstruction procedure using the full-sized TM, our method is free from the matrix inversion procedure, which is the most time-consuming throughout the whole process. Instead, we split the aberrant effects into two: focal distortion and spatial deformation. For the focal distortion, a phase mask (PM) compensating the phase disruption caused by the angle-dependent phase retardation of the FE lens is created from the minimally sampled TM. The lack of information involved with the reduction of the TM can be treated by an iterative interpolation method. Next, the spatial deformation is handled by attaining a geometric transformation for the space. By sequentially applying the PM and the inverse geometrical transformation to the taken images in order, both the focal distortion and the spatial deformation are corrected. The reconstructed image showed a contrast of 93.4%, and a signal-to-noise ratio (SNR) of 92.3% compared to the reconstruction results by the inversion method with the full-sized TM. Due to the use of the minimally sampled TM and no need for the inversion operation, this method took only 1.8% of processing time for the image correction, with almost no loss of imaging performance.

## Results

### Effect of angle-dependent phase retardations

**Figure 1:**
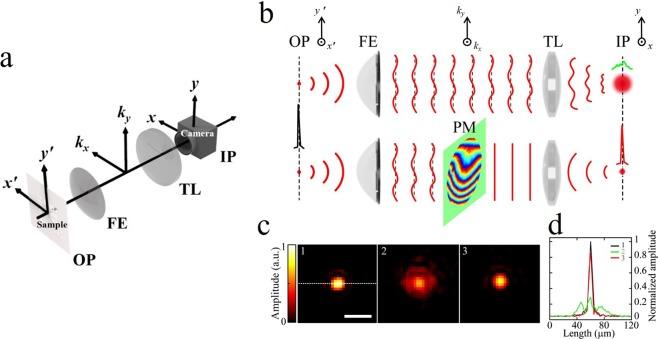
Principle of focal correction by the PM method. (**a**) Notations for planes and axes used in the text. (**b**) The wavefront disturbed by the aberration of the FE lens results in the focal broadening at IP (top). The correction of the wavefront by the PM results in the diffraction limited focal spot at IP (bottom). (**c**) 1: The single point generated at OP by the measured TM, 2: output point image generated at IP by the TM, and 3: output point image generated at IP after applying the PM. (**d**) Line profiles for 1 (black), 2 (green), and 3 (red) in. (**c**) OP: object plane. FE: fish-eye lens. PM: phase mask. TL: tube lens. IP: image plane. Scale bar: 20 μm.

Let us first consider the image formation of a target object located at the object plane (OP) through the imaging system equipped with the FE lens, as schematically shown in Fig. 1(a). When being illuminated with a plane wave, the object generates a wave, *E*_*OP*_(*x*′, *y*′), where (*x*′, *y*′) is the spatial coordinate for OP. When this wave propagates through the imaging system, the light disturbance resulting in the output image *E*_*IP*_(*x*, *y*) can be described by the transmission matrix *T* of the optical system as^17,19,20^

1 $${E}_{IP}(x,\,y)=T{E}_{OP}(x^{\prime} ,\,y^{\prime} ),$$

where (*x*, *y*) is the spatial coordinate for the image plane (IP) where the image sensor was placed. The simplest way to recover the original image from the light disturbance is applying the inverse of the TM *T*^−1^ on both sides of eq. (1). This is the general procedure which is valid for most situations regardless of the complexity of the light disturbance.

Due to the heavy calculation load associated with the huge-sized matrix manipulation for this process, we have developed an alternative method free from the matrix inversion, but which also works for systems with mild complexity, such as optical aberrations. When a single point is located at OP as an object, as shown in Fig. 1(b), it is transformed into the corresponding angular spectrum in *k*-space. Due to the angle-dependence phase retardations induced by the FE lens, the wavefront is disrupted and thus modified from its initial planar shape. As a result, after the inverse Fourier transform by the tube lens (TL), the wave cannot produce a sharp focus, resulting in the significant blurring at IP shown in Fig. 1(b) (top). In this situation, if we have information about the angle-dependent phase retardation, then we can compensate the phase disruption so that the wavefront becomes flat again in the *k*-space. Consequently, the wave can yield a sharp focus at IP, as presented in Fig. 1(b) (bottom).

In the TM frame work, we found that the effect of the TM associated with the angle-dependent phase retardations can be modeled as

2 $$T(x,\,y;\,{k}_{x},\,{k}_{y}){e}^{i({k}_{x}x^{\prime} +{k}_{y}y^{\prime} )}={e}^{i{\phi }_{ab}({k}_{x},{k}_{y})}{e}^{i({k}_{x}x+{k}_{y}y)}\equiv {E}_{T}(x,\,y;\,{k}_{x},\,{k}_{y}),$$

where (*k*_*x*_, *k*_*y*_) is the transverse components of wavevector *k*_0_ = λ/2π with the wavelength of light λ, ${E}_{T}(x,y;{k}_{x},{k}_{y})$ is the TM element, ${e}^{i({k}_{x}x^{\prime} +{k}_{y}y^{\prime} )}$ and ${e}^{i({k}_{x}x+{k}_{y}y)}$ are plane waves at OP and IP, respectively, and *φ*_*ab*_ (*k*_*x*_, *k*_*y*_) is the abnormal angle-dependent phase retardation caused by the optical system (See Supplementary Note 1). Through this simple model, the phase pattern which compensates for the phase disruption can be constructed as

3 $$P({k}_{x},\,{k}_{y})=F{[{E}_{IP}^{p}(x,y)]}^{\ast }={e}^{-i{\phi }_{ab}({k}_{x},{k}_{y})},$$

where *F* refers to a Fourier transform and ${E}_{IP}^{p}(x,\,y)$ is an output image formed at IP for a single point object located at OP as ${E}_{OP}^{p}(x^{\prime} ,\,y^{\prime} )=\delta (x^{\prime} ,\,y^{\prime} )$. Thus, the phase compensation pattern reduces simply to the conjugation of Fourier transform of a single point image at IP. We call this pattern a phase mask (PM) for the aberrant optical system. In this approximation, since the PM is position-independent, the phase compensation works for point objects located at any position at OP. Thus, the effect of the angle-dependent phase retardations imposed on output images of arbitrary objects can be canceled by applying *F*^−1^*PF* on the image *E*_*IP*_(*x*, *y*) taken at IP (See Supplementary Note 1 for more details).

### Construction of a PM

In experiments, a PM was constructed from a minimally sampled TM, which is a TM with a minimally sampled set of matrix elements, compared to a full-sized TM required for usual image reconstruction by the inversion operation. Under our experimental conditions, the size of the minimally sampled TM was 1/10 of that for the full-sized TM. For the minimally sampled TM construction, a transmission phase microscope equipped with two galvanometer mirrors (GMs) for angular scanning of an illumination beam was used. In the setup, a FE lens extracted from a wireless capsule-type endoscope was employed as a microscope objective. See more details for the setup in Supplementary Note 2.

We first measured a set of images transmitting through the FE lens system at IP as a function of the illumination angles for plane waves at OP. Typically 1,000 images were taken while scanning the illumination angle up to 0.14 NA at OP by the pair of GMs. These are output images for plane waves through the FE lens system, i.e., the TM elements *E*_*T*_(*x*, *y*; *k*_*x*_, *k*_*y*_). It should be noted that the full-sized TM requires 10,000 elements. Next, we need an image of a single point object for PM construction. Due to the uniformity of the interference signal^18^, the single point object and its image were numerically generated, rather than measuring a real spot. (See Methods and Supplementary Note 3 for more details.) First, a sharply focused spot was generated through the superposition of plane waves at OP, as shown in inset 1 in Fig. 1(c). The section profile is presented in Fig. 1(d). The full width at half maximum (FWHM) was measured at 5.68 ± 0.07 μm, which was determined by the illumination NA. Next, we also generated the corresponding image at IP by the superposition of the minimally sampled TM elements, as presented in inset 2 in Fig. 1(c). The point is significantly broadened due to the effect of the angle-dependent phase retardation.

The ratio of the peak intensity of an aberrated point image to that of an ideal point image affected only by the diffraction limit is known as Strehl ratio *S*, which is one of the simple measures for the degree of aberration. Since we already generated the diffraction-limited input point and the aberration-induced output point, *S* can be easily obtained by the ratio of the peak intensity of the output point to that of the input point. In our experiment, *S* was measured as 0.263, significantly lower than 0.8 the minimum Strehl ratio that optical systems should have to provide the near diffraction-limited images^24,25^. Thus, the distortion induced by the FL lens is in the strong aberration regime where the imaging performances, such as resolution and contrast, are significantly degraded.

In order to construct the suitable PM pattern, Fourier transforms were taken of the generated input and output point images. By comparing the phases of both the Fourier transformed images, the PM pattern could be determined as

4 $$P({k}_{x},\,{k}_{y})=F[{E}_{OP}^{p}(x^{\prime} ,\,y^{\prime} )]/F[{E}_{IP}^{p}(x,\,y)]={e}^{-i{\phi }_{ab}({k}_{x},{k}_{y})}.$$

For practical reasons, this is more accurate than the result obtained from eq. (3). By comparing the two Fourier transforms, the unwanted phase modulation that might be imposed on the input plane waves ($c({k}_{x},\,{k}_{y})$ in Materials and Method) at the OP side can be safely rejected. As a result, only the aberrant effect can be extracted with eq. (4).

Since the points were numerically generated with a limited number of waves, the information on each angular spectrum is also incomplete. This results in a substantial number of empty pixels in the PM area. Due to the slowly varying nature of the angle-dependent phase retardation of the FE lens, we can iteratively fill in the missing information so that the PM has a smooth surface over the whole area (See Supplementary Note 4 for more details). The final PM pattern constructed with eq. (4) and the iteration method is shown in Fig. 1(b) (Also see Supplementary Fig. 4). From the final PM pattern, the aberration strength, which is defined as the root mean square (RMS) aberrated phase deviation in the *k*-space compared to the flat phase distribution of an ideal system, was measured as 1.66 radian. The value varied from 1.40 to 1.80 depending on the lens alignment, but always exceeded the range of intermediate aberration strength^26,27^. This is another evidence that our system is in the strong aberration regime.

With the information about the PM, we then applied *F*^−1^*PF* to the broadened output image for the focus correction. The result is shown in inset 3 in Fig. 1(c). The focal spot became sharp, similarly to the original shape. For comparison, the cross-section profiles for the spots are presented in Fig. 1(d). After the correction, the FWHM for the peak was measured to be 5.71 ± 0.09 μm, which was fairly close to that of the original object.

### Correction of spatial distortion by geometric transformation

**Figure 2:**
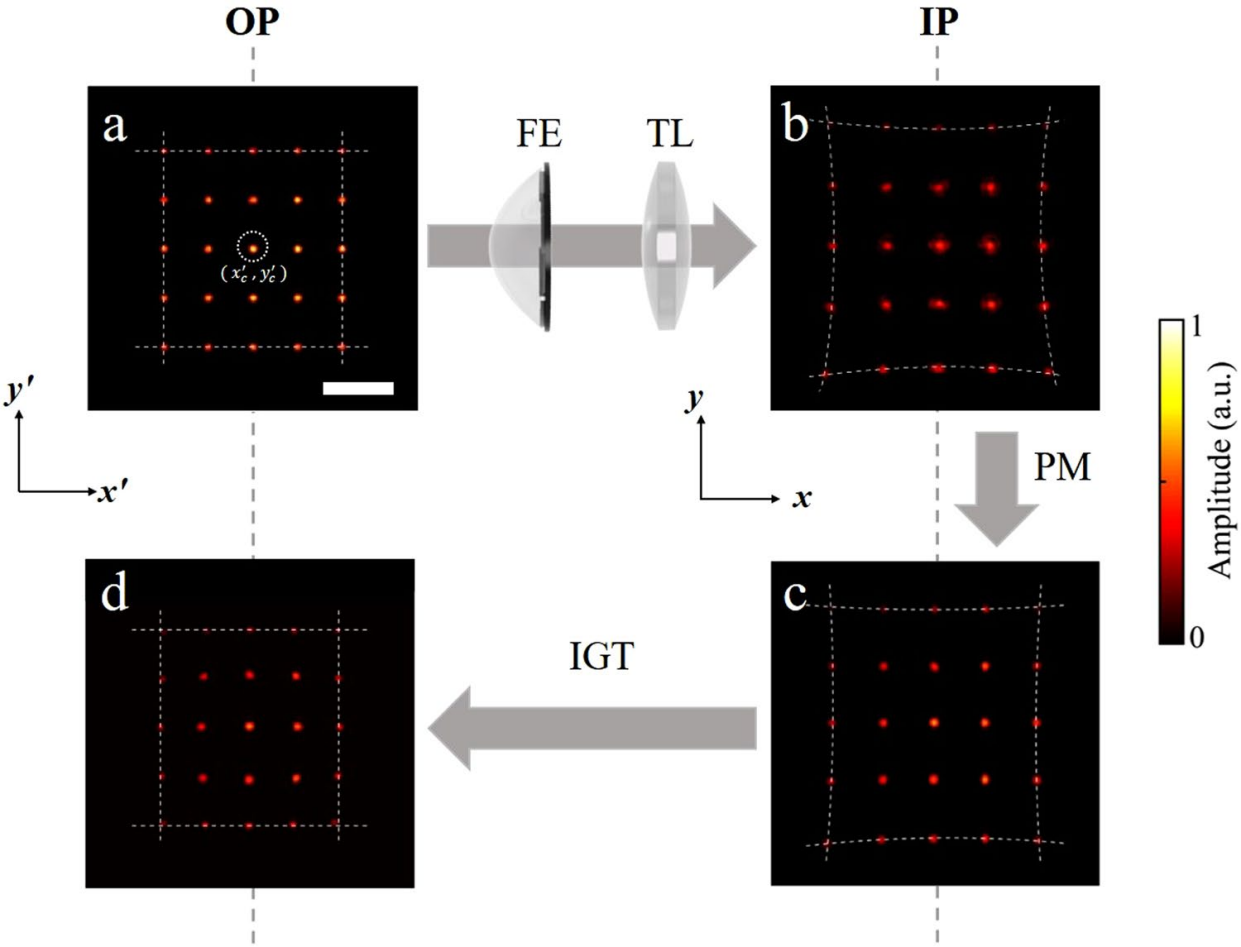
Correction for the spatial distortion using the LM pattern. (**a**) Formation of points in LM pattern generated by the plane waves measured at OP. (**b**) Corresponding LM pattern after transmitting through the FE lens system, which was generated by the TM elements measured at IP. (**c**) After applying the PM method to (**b**) for the focal correction. (**d**) After the spatial correction by IGT. Scale bar: 100 μm.

The focal distortion that associated the angle-dependent phase shifts was corrected by introducing a PM which could cancel the effect of phase retardation. After the focal correction, however, the distortion involved with spatial deformation still resided in the image, as shown in Fig. 2. This was due to the variation of the spatial magnification along the radial direction. In order to correct this distortion, we compared the two spaces in OP and IP by creating a landmark (LM) pattern consisting of 5 × 5 points distributed in a square form. These points were generated in OP space, one by one, by the superposition of the angular plane waves as was done for the PM construction. First, we generated the LM pattern at OP as shown in Fig. 2(a). The points in the LM could be expressed as ${P^{\prime} }_{i}={[{x^{\prime} }_{i},{y^{\prime} }_{i}]}^{T}$, where ${P^{\prime} }_{i}$ denotes the *i*-th point located at $({x^{\prime} }_{i},\,{y^{\prime} }_{i})$ and []^*T*^ is a matrix transposition. Since all of the points are generated at OP, there’s no distortion in the LM pattern with ${P^{\prime} }_{i}$ s, as shown in Fig. 2(a). As a response of the LM pattern to the FE lens system, each of the corresponding points at IP was also generated by the superposition of the measured minimally sampled TM elements. Once ${P^{\prime} }_{i}$ s pass through the FE lens system, they simultaneously suffer from focal distortion and spatial distortion, as presented in Fig. 2(b). The change in the length scale is due to the overall magnification of the FE-lens system from OP to IP. Aside from the magnification change, the shifts of the points from the original positions caused by the spatial distortion are observed at IP. After the correction of the focal distortion by the PM method, the sharp focus was recovered almost over the entire field of view, as shown in Fig. 2(c). Note that the image is still magnified because the points are still in IP space. The locations of the points at IP can be expressed as ${P}_{i}={[{x}_{i},{y}_{i}]}^{T}$, where *P*_*i*_ denotes the point at IP located at $({x}_{i},{y}_{i})$. In order to relate all ${P^{\prime} }_{i}$ s and *P*_*i*_ s, we used the Brown-Conrady distortion equation model^28–30^ which describes the spatial distortion based on a radial and a tangential deformation components. In the picture of the Brown-Conrady model, each point pair of ${P^{\prime} }_{i}$ and *P*_*i*_ is related as

5 $$[\begin{array}{c}{x}_{i}\\ {y}_{i}\end{array}]=(1+{k}_{1}{r}_{i}^{2}+{k}_{2}{r}_{i}^{4})[\begin{array}{c}{x^{\prime} }_{i}\\ {y^{\prime} }_{i}\end{array}]+[\begin{array}{c}2{p}_{1}{x^{\prime} }_{i}{y^{\prime} }_{i}+{p}_{2}({r}_{i}^{2}+2{{x^{\prime} }_{i}}^{2})\\ 2{p}_{2}{x^{\prime} }_{i}{y^{\prime} }_{i}+{p}_{1}({r}_{i}^{2}+2{{y^{\prime} }_{i}}^{2})\end{array}].$$

Here, ${r}_{i}=\sqrt{{({x^{\prime} }_{i}-{x^{\prime} }_{c})}^{2}+{({y^{\prime} }_{i}-{y^{\prime} }_{c})}^{2}}$ is a radial distance from the origin denoted by $({x^{\prime} }_{c},\,{y^{\prime} }_{c})$ at OP, and *k*_1_, *k*_2_, and *p*_1_, *p*_2_ are the coefficients representing the radial and the tangential distortions, respectively. These coefficients are determined by fittings using the positions of ${P^{\prime} }_{i}$ s and ${P}_{i}$ s in the LM pattern. Equation (5) together with the determined coefficients describes the geometric transformation (GT) caused by the distortion when the spaces are transferred from OP to IP. Thus, the image correction can be performed by the inverse geometric transformation (IGT), which makes the curved space at IP flat, as it is at OP. Since the IGT transfers the IP space to the OP space, the magnification change is automatically taken into account so that the final LM image has the same length scale as that of the original LM pattern, as shown in Fig. 2(d).

### Verification for correction method using PM and LM

**Figure 3:**
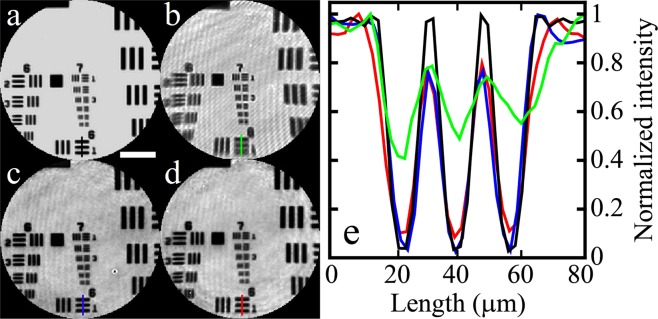
The comparison of the image reconstruction. (**a**) Reference image of a USAF target taken by the separate imaging system. (**b**) Intact distorted image generated by averaging 100 intensity images. (**c**) Reconstructed image by the inversion of the full-sized TM. (**d**) Reconstructed image by the PM-LM method. (**e**) Line profiles for the structures in the USAF target across the black, green, blue and red lines in (**a**–**d**), respectively. Scale bar: 100 μm.

Before evaluating the performance of our correction method, a reference object image was acquired for comparison. As the reference, an image of a United States Air Force (USAF) resolution target was taken by a separate imaging system equipped with standard imaging optics and an LED illumination. As shown in Fig. 3(a), the reference image has almost no aberration over the whole field of view. For fair comparison, the spatial resolution of the reference imaging was tuned to the same as that of the FE lens system by adjusting the size of the aperture located at the Fourier plane of the imaging lens. Since the USAF target is a pure intensity object, the background noise was cleaned by rejecting the intensity variation smaller than a certain threshold level.

Next, an image of the same USAF target was taken by the FE lens imaging system. For this imaging, 100 images were taken at various illumination angles covering 0.11 NA. Then, all of the taken images were processed into intensity images and were averaged to produce a bright-field equivalent image. Since the NA of the FE lens is 0.14, the final imaging NA is 0.25 after the averaging. Due to the aberration, the image shows the focal and spatial distortion as shown in Fig. 3(b). Although the image has a sharp focus around the center region, similar to the reference image in Fig. 3(a), it loses the focus off the center region. The focal distortion worsens along the radial direction from the center to the edge. The image was blurred out significantly, particularly in the bottom area. The asymmetry in the tendency of the focal distortion was due to the slight misalignment of the FE lens in tilt. In addition to the focal distortion, the image seems to have curved space, i.e., it is stretched along the two diagonal directions more than along the vertical and horizontal directions. This is a spatial distortion attributed to the pincushion aberration.

For the aberration correction, a full-sized TM for the FE lens was measured prior to further image processing. For the TM measurement, 10,000 images for the empty plane without the USAF target were acquired while scanning the incident angle. The angular coverage of the scanning corresponded to the maximum NA supported by the FE lens, 0.14 NA. After the TM measurement, we reconstructed the object images using the standard TM inversion method^17,19,20,23^. We applied *T*^−1^ to all the taken USAF target images and inverted the distortion. Then, the reconstructed images were synthesized by the synthetic aperture technique^22,31^ in order to produce the final object image, as shown in Fig. 3(c) (See Methods). After the synthesis, the achieved imaging NA was enhanced, resulting in 0.25 NA. After this reconstruction, the focal plane became flat up to the edge region so the structures in element 1 in group 6, which were mostly defocused in the distorted image in Fig. 3(b), could be well resolved. In addition to the focal correction, the spatial deformation caused by the pincushion distortion was also corrected. This is because the TM-based reconstruction method transformed the plane of interest from IP to OP and reversed all distortions.

We also reconstructed the object image using the proposed method. The minimally sampled TM was constructed with 1,000 elements by choosing one out of every ten elements for the full-sized TM. After the focal correction by applying the PM in the *k*-space of the object images, the remaining spatial distortion could be corrected by the IGT determined by the LM pattern, which was also generated by the minimally sampled TM. After the corrections, all of the reconstructed images were synthesized for the final object image as presented in Fig. 3(d). The image is uniformly focused as well as the spatial distortion is corrected over the entire field of view, similar to that reconstructed by the full-sized TM. Figure 3(e) shows the line profiles of the structures in element 1 in group 6. Black (reference), green (intensity averaging), blue (TM inversion), and red (PM-LM) represent the profiles along the lines in Fig. 3(a–d), respectively. As shown with the green line, the structures in the edge region were significantly blurred in the intact image. However, TM inversion and PM-LM methods show sharp profiles, revealing the structures clearly. The image contrast and the SNR of the PM-LM reconstruction were measured as 93.4% and 92.3%, respectively, compared to those of the full-sized TM inversion method.

Our method using the PM and LM shows almost the same performances as those with the full-sized TM. But, due to the absence of the matrix operation involved with *T*^−1^, the reconstruction time is significantly reduced. In our experiment, the reconstruction of the individual object images by applying the inverse of the TM took 47.72 seconds and the aperture synthesis for those images took an additional 0.50 seconds. Thus, the TM method is far behind the real-time imaging of an object. In the PM-LM method, in contrast, the image reconstruction is performed by sequentially applying the PM in the *k*-space and the IGT in the position space. The processing time for applying the PM and the IGT took 0.22 seconds and 0.16 seconds, respectively, for each. The image synthesis, which is the most common for both the TM and PM-LM methods, takes 0.50 seconds. Thus, the PM-LM method took 0.88 seconds in total, which is 1.8% of and thus 55 times faster than the full-sized TM method. For more details about the assessment for the processing times, see Supplementary Note 5.

### Image correction for a biological sample

**Figure 4:**
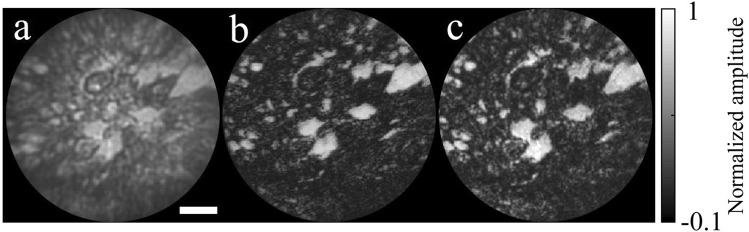
Image reconstruction for a skin tissue of a rat. (**a**) Simple average of the taken images by the FE lens system. (**b**) Reconstructed image by the inversion of the full-sized TM (**c**) Reconstructed image by the PM-LM method. Scale bar: 100 μm, color bar: amplitude (arbitrary unit).

The capability of our method for imaging biological samples was also demonstrated. In order to do this, we carried out test imaging with a mouse skin tissue as a biological sample. The skin tissue was extracted from a nude mouse (aged five weeks) and sliced by a cryostat microtome with a thickness of 10 μm. Figure 4(a) shows the tissue image taken by the FE lens system, but the image was generated by a simple averaging of the individual images. Significant focal and spatial distortion was introduced by the FE lens. Next, both the reconstruction methods were applied to the same taken images. As a comparison, the reconstructed image by the inverse of the full-sized TM is presented in Fig. 4(b). As expected, the detailed structures are clearly visualized up to the edge region. For our method, PM and LM patterns were generated using the minimally sampled TM with 10% of the elements of the full-sized TM. After sequentially applying the PM and IGT, the reconstructed image was obtained, as shown in Fig. 4(c). The structural details show fairly good agreement with those by the inverse of the full-sized TM. Both the TM and PM-LM reconstructions show enhanced SNR and image contrast compared to those of the simple averaging. Thus, we can confirm that our method is applicable to the imaging of biological samples and is able to provide comparable performances for image reconstruction with the inversion method using the full-sized TM.

## Discussion

As confirmed in Figs. 3 and 4, the image reconstruction by the PM-LM method provides an outstanding performance in terms of processing time and also offers comparable image quality to that by the full-sized TM. The TM inversion method shows a slightly better image quality than our method, because it is able to utilize more information regarding the optical system. However, the result is seriously influenced by the size of the TM. Even with the reduction by half in the TM elements, most of the structures were covered by significant noise (See Supplementary Note 6). Thus, acquiring a huge data set is essential for the TM inversion method, consequently, the concomitant increase in the processing time significantly ruins the benefit of the slight increase in the image quality.

In contrast, our method can take the advantage of the simple procedure due to the interpolation capability for the missing information caused by the reduced sampling, while keeping the image quality comparable to that by the full-sized TM. It was found that the constructed PM distribution remained the same with the reduced sampling down to 10% of the full-sized TM (See Supplementary Note 4). This means that the reconstruction result is almost unaffected with this minimally sampled TM. With lower than 10%, the reconstruction quality drops due to the failure of the interpolation for the PM construction. The preservation of the reconstruction performance is attributed to the fact that the phase value in the PM varies so slowly that the interpolation process works up to 10% reduced sampling compared to the full-sized TM. If the sampling ratio drops below 10%, the interpolation starts to fail to fill the missing phase values in the PM construction. Since the PM patterns vary from system to system, 10% sampling ratio is a specific value for our system. Generally, the minimum sampling ratio for the TM, while keeping the reconstruction performance, depends on the complexity of the angle-dependent phase retardation of the optical system. We performed additional experiments by introducing various types of aberration using an aspheric lens. Although with the different aberration effects induced by different optical configurations, our method consistently provided the reconstruction performances similar to those by the inversion of the full-sized TM. This confirmed that our method can be applied to more general situations. However, the minimal sampling ratio of the TM for the PM creation increased with the complexity of the aberration due to the requirement for the success of the iterative interpolation (See Supplementary Note 7).

Our method requires a minimal set of TM elements for PM construction and LM creation. This reduced both the measurement and processing time for the pre-calibration procedures associated with the TM preparation. Another cut in processing time came from the inversion-free nature of the PM correction. Since the focal correction was performed by applying the PM to the *k*-space of the output images, it could avoid the time-consuming matrix multiplication operation. This posed a clear advantage for the proposed method in reconstruction speed. Even with the image recovery 55 times faster than the inversion method with the full-sized TM, our method still preserved the reconstruction performances, i.e., imaging SNR and contrast, at the level of 92.3% and 93.4%. Thus, our method could take all of the benefits from the simplified procedures, such as the minimal sampling of the TM and the absence of the TM inversion, while maintaining the whole point of using the TM, i.e., having the deterministic connection between the input and output planes.

With all the advantages in the image processing without scarifying the image quality, our method can offer the high-speed and accurate correction for the system’s aberration. This will facilitate high-resolution and high-precision measurements for the target objects using optical systems equipped with strongly aberrant lenses due to the limit of small space and light weight, such as wireless endoscopes, portable imaging devices, etc.

## Materials and Methods

### Generation of single spots at OP and IP

We generated a single point object ${E}_{OP}^{p}(x^{\prime} ,\,y^{\prime} )$ at one position at OP by a superposition of the 1,000 input plane waves with the same illumination angles with those of the minimally sampled TM elements. In this construction, all input fields were added in phase at the center position as, ${E}_{OP}^{p}(x^{\prime} ,\,y^{\prime} )=\sum _{{k}_{x},{k}_{y}}^{N}c({k}_{x},\,{k}_{y}){e}^{i({k}_{x}x^{\prime} +{k}_{y}y^{\prime} )}$, *c*(*k*_*x*_, *k*_*y*_) is the superposition coefficients with which the generated point is located at $(x^{\prime} ,\,y^{\prime} )$, $|c({k}_{x},{k}_{y})|=1$ for all (*k*_*x*_, *k*_*y*_), and *N* is the number of the taken images, which was 1,000 in this experiment.

The output image corresponding to this virtual single point can also be generated by the superposition of the TM elements with the same superposition coefficients with those for the input single point as, ${E}_{IP}^{p}(x,\,y)\,=$ $\sum _{{k}_{x},{k}_{y}}^{N}c({k}_{x},\,{k}_{y}){E}_{T}(x,\,y;\,{k}_{x},\,{k}_{y})$, where *E*_*T*_ is the TM elements in eq. (2).

### Image synthesis

We take multiple object images with various oblique illuminations. The distortion on these images are removed by our correction method as described in the main text. After correction, all of the taken images are synthesized in order to produce the final object image. With the oblique illumination with a transverse wavevector $({k}_{x}^{ill},\,{k}_{y}^{ill})$ at OP, the object spectrum is shifted as

$${A}_{s}({k}_{x},\,{k}_{y})=P({k}_{x},\,{k}_{y})\tilde{O}({k}_{x}-{k}_{k}^{ill},\,{k}_{y}-{k}_{y}^{ill}),$$

where ${A}_{s}({k}_{x},\,{k}_{y})$ is Fourier transform of the measured image, $P({k}_{x},\,{k}_{y})$ is the pupil function of the FE lens with value of unity when $\sqrt{{k}_{x}^{2}+{k}_{y}^{2}}\le {k}_{0}\,{\rm{NA}}$ and zero elsewhere, and $\tilde{O}({k}_{x}-{k}_{k}^{ill},{k}_{y}-{k}_{y}^{ill})$ is the shifted object spectrum. Then, the object spectrum in the unshifted coordinate is obtained by shifting the measured Fourier transform by $({k}_{x}^{ill},\,{k}_{y}^{ill})$. Thus, the Fourier transform of the synthesized image can be constructed as a superposition of the shifted individual transforms as

$${A}_{syn}({k}_{x},\,{k}_{y})=\sum _{{k}_{x}^{ill},{k}_{y}^{ill}}{A}_{s}({k}_{x}+{k}_{x}^{ill},{k}_{y}+{k}_{y}^{ill})=\sum _{{k}_{x}^{ill},{k}_{y}^{ill}}P({k}_{x}+{k}_{x}^{ill},{k}_{y}+{k}_{y}^{ill})\tilde{O}({k}_{x},\,{k}_{y}).$$

The final synthesized object image can then be obtained by an inverse Fourier transform of ${A}_{syn}({k}_{x},\,{k}_{y})$.

### Skin tissue preparation

All animal experiments were performed according to a protocol approved by our Institutional. Animal Care and Use Committee (Korea-2017-0156).

The skin tissue was extracted from a nude mouse with an age of five weeks old. The tissue was cut into a block with a dimension of 5 × 5 × 1 mm^3^, washed gently in ice-cold phosphate-buffered saline (PBS, pH 7.2) solution, and then washed in isotonic (0.25 mol/L) solution. The block of tissue was immersed into solution of 4% paraformaldehyde at temperature of 4 °C for 24 hours and kept in a solution of 30% sucrose at 4 °C for overnight for fixation. After that, the tissue block was immersed in an optimal cutting temperature (OCT) compound, snap-frozen in liquid nitrogen, and stored at −80 °C. The block of frozen tissue was sliced with a thickness of 10 μm using a microtome (CM3050S, Leica), then transferred onto a slide glass. The slide glass was heated gently on a slide warmer (40 °C) until the slice was spread and flattened out. Finally, the tissue slice was mounted on a 24 × 24 mm^2^ coverslip using a mounting medium.

## Supplementary information

Inversion-free image recovery from strong aberration using a minimally sampled transmission matrix
