# Supplementary material for: Inversion-free image recovery from strong aberration using a minimally sampled transmission matrix
Source: Sci Rep. 2019 Feb 4;9:1206. doi: 10.1038/s41598-018-38027-y (PMC6361891; doi:10.1038/s41598-018-38027-y)
Supplement: Supplementary file 1 — Inversion-free image recovery from strong aberration using a minimally sampled transmission matrix [file 41598_2018_38027_MOESM1_ESM.docx]

**Inversion-free image recovery from strong aberration using a minimally sampled transmission matrix**

Kwanjun Park^1,†^, Taeseok Daniel Yang^2,†^, Hyung-Jin Kim^1^, Taedong Kong^1^, Jung Min Lee^3^, Hyuk Soon Choi^3^, Hoon Jai Chun^3^, Beop-Min Kim^1,2^, and Youngwoon Choi^1,2,*^

*^1^Department of Bio-convergence Engineering, Korea University, Seoul 02841, South Korea*

*^2^School of Biomedical Engineering, Korea University, Seoul 02841, South Korea*

*^3^Department of Gastroenterology and Hepatology, Korea University, Seoul 02841, South Korea*

*^†^Theses authors contributed equally to this work.*

*^*^Corresponding author: youngwoon@korea.ac.kr*

**Supplementary Note 1**

**Correction for focal distortion by a phase mask**

We consider the image formation of a target object between the object plane (OP) and the image plane (IP) by the imaging system equipped with the FE lens as shown in Supplementary Fig. 1. When being illuminated with a plane wave, the object generates a wave, *E_OP_*(*x′*,*y′*), where (*x′*,*y′*) is the spatial coordinate for the OP. When this wave propagates through the imaging system, the way of light disturbance resulting in the output image *E_IP_*(*x*,*y*) at IP can be described by the transmission matrix *T* of the FE lens system as [^1-3^](#_ENREF_1)

$E_{IP}(x,y)=TE_{OP}(x',y')$. (1)

where (*x*,*y*) is the spatial coordinate for the IP. The simplest way to recover the original image from the light disturbance is applying the inverse of the transmission matrix $T^{-1}$ on both sides of Eq. (1). This is the general expression which holds for most situations regardless of the complexity of the light disturbance.

Due to the heavy calculation load associated with the huge-sized matrix manipulation for this process, here we will develop an alternative method which also works for the system with mild complexity such as optical aberration. From the wave nature of the light, *E_OP_*(*x′*,*y′*) can be thought to form the object image with various angular plane waves. The electric field for the object image then can be expressed as;


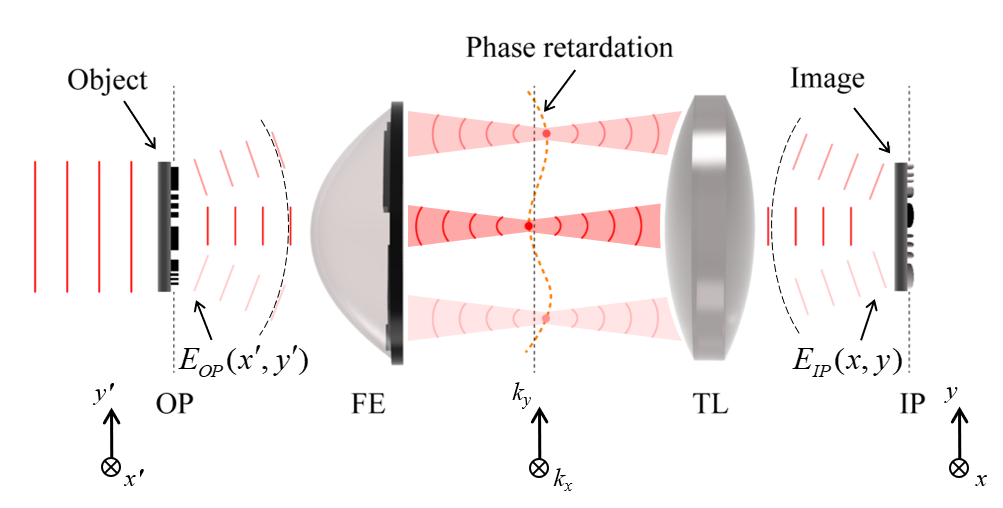


**Supplementary Figure 1.** Schematic for the image formation through the FE lens imaging system. The phase retardation makes it difficult for the system to form an image with sharp focus.

. (2)

Where (*k_x_*, *k_y_*) is the components of wavevector *k* = λ/2π with the wavelength of light λ, *A_s_*(*k_x_*, *k_y_*) is the angular spectrum of the object at the OP with a magnitude |*A_s_*(*k_x_*, *k_y_*)| and a phase *ϕ_s_*(*k_x_*, *k_y_*). Note that *A_s_*(*k_x_*, *k_y_*) = *F*[*E_OP_*(*x′*,*y′*)] where *F* is a Fourier transform. The transmission matrix describing the characteristic of the light transmission from OP to IP can be written as *T*(*x*,*y*;*k_x_*,*k_y_*) by which each plane wave $e^{i(k_{x}x'+k_{y}y')}$ is transformed into *E_T_*(*x*,*y*;*k_x_*,*k_y_*), where *E_T_*(*x*,*y*;*k_x_*,*k_y_*) is the element of the transmission matrix for the imaging system. At the IP, the electric field of the delivered object image *E_IP_*(*x*,*y*) can be expressed as a superposition of the multiple transmission matrix elements as,

$\begin{matrix} E_{IP}(x,y)=TE_{OP}(x',y') \\ \text{ }=\iint A_{s}(k_{x},k_{y})T(x,y;k_{x},k_{y})e^{i(k_{x}x'+k_{y}y')}dk_{x}dk_{y}=\iint A_{s}(k_{x},k_{y})E_{T}(x,y;k_{x},k_{y})dk_{x}dk_{y}. \end{matrix}$ (3)

Note that Eq. (3) is another expression of Eq. (1). Thus this is also a general expression valid for the effect of light transmission regardless of the degree of light disturbance. As a special case, for the ideal optical system with no aberration, $T(x,y;k_{x},k_{y})e^{i(k_{x}x'+k_{y}y')}=e^{i(k_{x}x+k_{y}y)}$. Thus the object image at OP, which is represented by (*x′*,*y*′)-coordinate, is transferred to IP represented by (*x*,*y*)-coordinate where the perfectly duplicated image is formed.

With an aberrant optical system, the effect of the light transmission can be assumed to be phase retardations depending on the wavevector components (*k_x_*, *k_y_*). Thus, the transmission matrix for the aberrant optical system is modeled as , where *ϕ_ab_* (*k_x_*, *k_y_*) is the abnormal angle-dependent phase retardation caused by the optical system. With this simple modeling the output object image is described by

. (4)

Then the Fourier transform of the output image becomes

. (5)

If a single point is used as the input object as $E_{OP}^{p}(x',y')=\delta(x',y')$, its angular spectrum $A_{s}^{p}(k_{x},k_{y})=1$ for all (*k_x_*, *k_y_*), and the Fourier transform of the corresponding output field will be given as by Eq. (5). Thus, the phase retardation caused by the optical aberration can be obtained by the Fourier transform of the output image of a single point. From this information we can construct a phase mask (PM) pattern as

. (6)

Then the effect of the angle-dependent phase retardation can be canceled by applying *F*^-1^*PF* on the output object image *E_IP_*(*x*,*y*) .

**Supplementary Note 2**

**Experimental setup**

The experimental setup is schematically presented in Supplementary Fig. 2. The optical configuration is based on a standard-type transmission phase microscope with an off-axis detection scheme. A He-Ne laser (Thorlabs: HNL210L) with a wavelength of λ = 632.8 nm is used as a light source of which output is divided into two optical paths by beam splitter 1 (BS1). One beam is sent to the sample arm for illumination and the other to free space to form a clean reference. In the sample path, after being expanded by a beam expander (BE1), the beam is steered by two galvanometer scanning mirrors (GM*_x_* and GM*_y_*; Thorlabs: GVS211). The two scanning mirrors are positioned at the front conjugate planes of the sample plane so that the incident angle impinging onto a sample is varied by the mirror scanning. A fish-eye (FE) lens, which was detached from a capsule-type wireless endoscope, is used as an objective lens to image an object in conjunction with a tube lens (TL). The overall numerical aperture (NA) of the FE lens imaging system is 0.14. The reference beam is expanded by another beam expander (BE2) to a plane wave with a dimension covering the entire detection area. The reference beam is then combined with the sample beam at BS2 at an angle to generate an off-axis interference image. The interference image is captured by a camera (Point Grey: FL3-U3-13Y3M-C). The camera exposure is synchronized with the GMs such that multiple images are recorded while scanning the illumination angle of the sample beam. Typically sample images are obtained at 100 fps, which is the limit of the camera acquisition rate. The acquired interferograms are processed into complex field images carrying both the amplitude and phase information of the samples [^4^](#_ENREF_4).


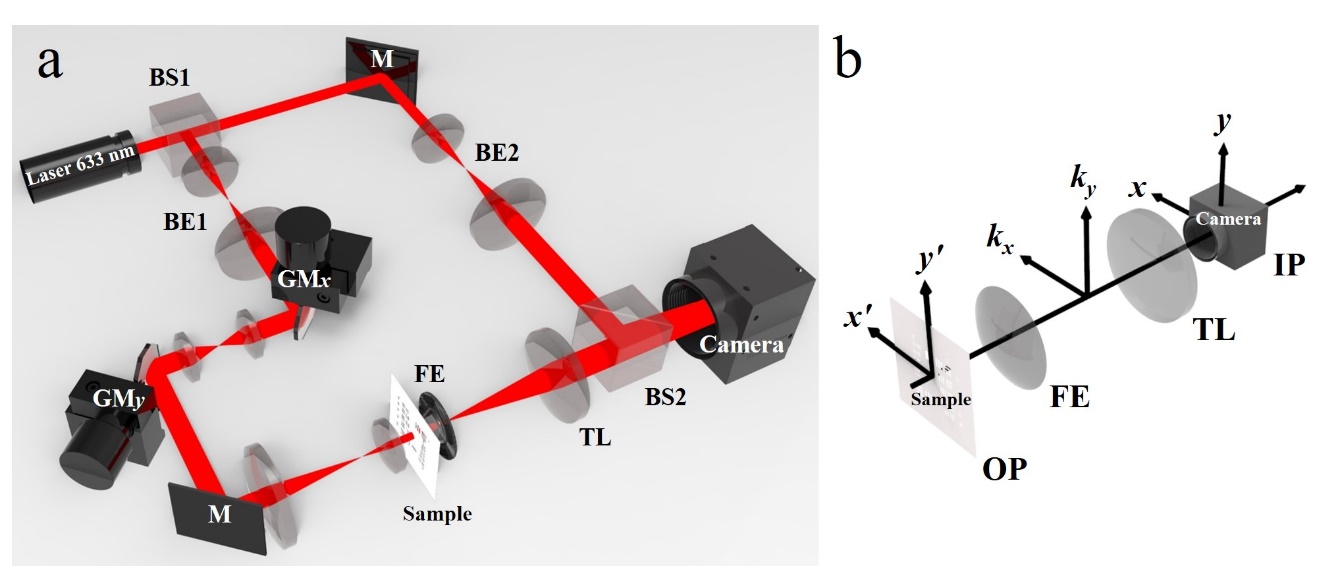


**Supplementary Figure 2.** Experimental schematic for the FE lens system. (a) BS1 and BS2: beam splitters, GM*x* and GM*y*: galvanometer mirrors, BE1 and BE2: beam expanders, FE: fish-eye lens, TL: tube lens, M: mirrors. (b) Notations for planes and axes used in the text. OP: object plane, IP: image plane.

**Supplementary Note 3**

**Generation of a single point object at OP and its image at IP**

Supplementary Figure 3 shows the schematic for the generation of a PM pattern in the experiments. We first measure a set of images of plane angular waves at OP. For the analysis, 1,000 images were taken while scanning the illumination angle by the GMs corresponding to 0.14 NA. These images are the input plane waves $e^{i(k_{x}x'+k_{y}y')}$ at OP. Next, we measure another set of images transmitted through the FE lens system at IP with the same illumination angles with those of the input waves. These are output images for the FE lens system, i.e., the TM elements *E_T_*(*x*,*y*;*k_x_*,*k_y_*). Due to the limited dynamic range of a camera, a single point was numerically generated rather than imaging a real spot at OP. As shown in Supplementary Figure 3, we generated a single point at one position of OP by a superposition of the input field images. In this construction, all input fields were added in phase at the center position as,

$E_{OP}^{p}(x',y')=\sum_{k_{x},k_{y}}^{N} c(k_{x},k_{y})e^{i(k_{x}x'+k_{y}y')}$, (7)

where $c(k_{x},k_{y})$is the superposition coefficients with which the generated point is located at $(x',y')$, $\left|c(k_{x},k_{y})\right|=1$ for all (*k_x_*,*k_y_*), and *N* is the number of the taken images. The full with at half maximum (FWHM) of the generated spot at OP was measured as 5.68±0.07 μm as shown in Fig. 1(c) in the main text.

The output image corresponding to this virtual single point can also be generated by the superposition of the TM elements, as shown in Supplementary Figure 3, with the same superposition coefficients with those for the input single point as,

(8)

where *E_T_* is the TM elements. The generated point is significantly broadened due to the effect of the angle-dependent phase retardation caused by the term as presented in Fig. 1(c) in the main text. To construct the suitable PM pattern for correction of the focal broadening, Fourier transforms of the input and output images were taken. By comparing the phases of both the angular spectra, the correction PM pattern could be obtained as

. (9)

Then we applied *F*^-1^*PF* to the broadened output image for the focus correction. The result is shown in inset 3 in Fig. 1(c) in the main text. After the correction the FWHM for the peak was measured as 5.71±0.09 μm, which is fairly close to that of the original object.


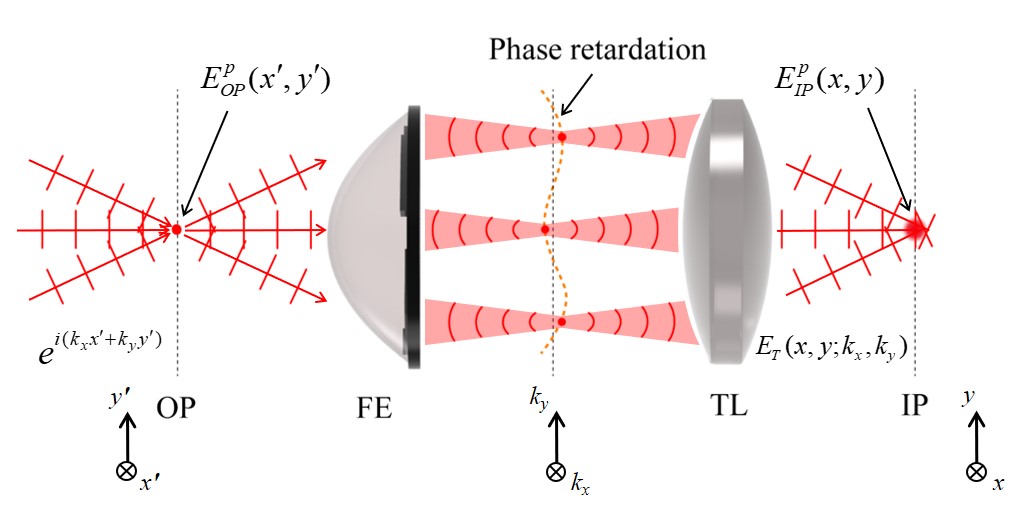


**Supplementary Figure 3.** Generation of the single point object and its output image. The input point is generated at OP by the in-phase superposition of the measured plane waves. The corresponding output point image is generated at IP with the TM elements with the same superposition coefficients with those of the input point. The output point loses its focus due to the effect of the aberration induced by the FE lens.

**Supplementary Note 4**

**Iterative interpolation for a PM with the minimally sampled TM**

In the inversion method, the quality for the image reconstruction highly depends on the extent of the TM. The NA of the reconstruction is determined by the angular coverage of the TM measurement and the reconstruction SNR depends on the number of TM elements within the angular coverage [^5^](#_ENREF_5). Thus, the huge amount of data is required for high quality image reconstruction in the inversion method. In the same sense, the performance of a PM construction is also determined by the TM measurement. Since the virtual point for the PM construction is created by the superposition of the TM elements, each TM element corresponds to each pixel of the PM in the *k*-space. Thus, the angular coverage of the TM measurement limits the NA of the PM construction and the number of TM elements determines the number of phase values in the PM area.

In our experiments, we measured 10,000 images for the full-sized TM construction with which we could obtain the reasonable reconstruction quality within the available NA of the FE lens. If we use the full-sized TM with 10,000 elements for the PM construction, we obtain the phase distribution as shown in Supplementary Fig. 4(a). The irregular phase distribution caused by the angle-dependent phase retardation can be observed. Even with the full-sized TM, phase values on some pixels are still undetermined due to the lack of TM elements for completely filling the PM area. These empty pixels can cause reconstruction artifact when applying the focal correction, thus we iteratively fill the missing points so that the PM has a smooth surface as shown in Supplementary Fig. 4(b). Since the phase varies gradually in the PM due to the slowly varying phase retardation of the FE lens, the iterative filling for the missing information also works for lower sampling ratio of the TM than 10,000 images. To explore this point, we intentionally reduced the sampling ratio from the full-sized TM by 50 %, 30 %, and 10 % while retaining the measurement NA. And then we constructed the PM by creating the virtual point with the lower sampled set of TM elements for each case. As shown in Supplementary Figs. 4(c)-(e), more pixels became empty with the reduction of the sampling ratio for the TM. In contrast, after applying the iterative interpolation method, the PMs have smooth surfaces for all the cases as shown in Supplementary Figs. 4(f)-(h). With 10 % sampling ratio, the phase distribution started to be slightly degraded due to the substantial loss of information. From this investigation, we concluded that a PM for the reasonable reconstruction can be created with only 10 % of data compared to the full-sized TM. The systematic evaluation for the performance of the PM by the minimally sampled TM elements will be discussed in Supplementary Note 6.


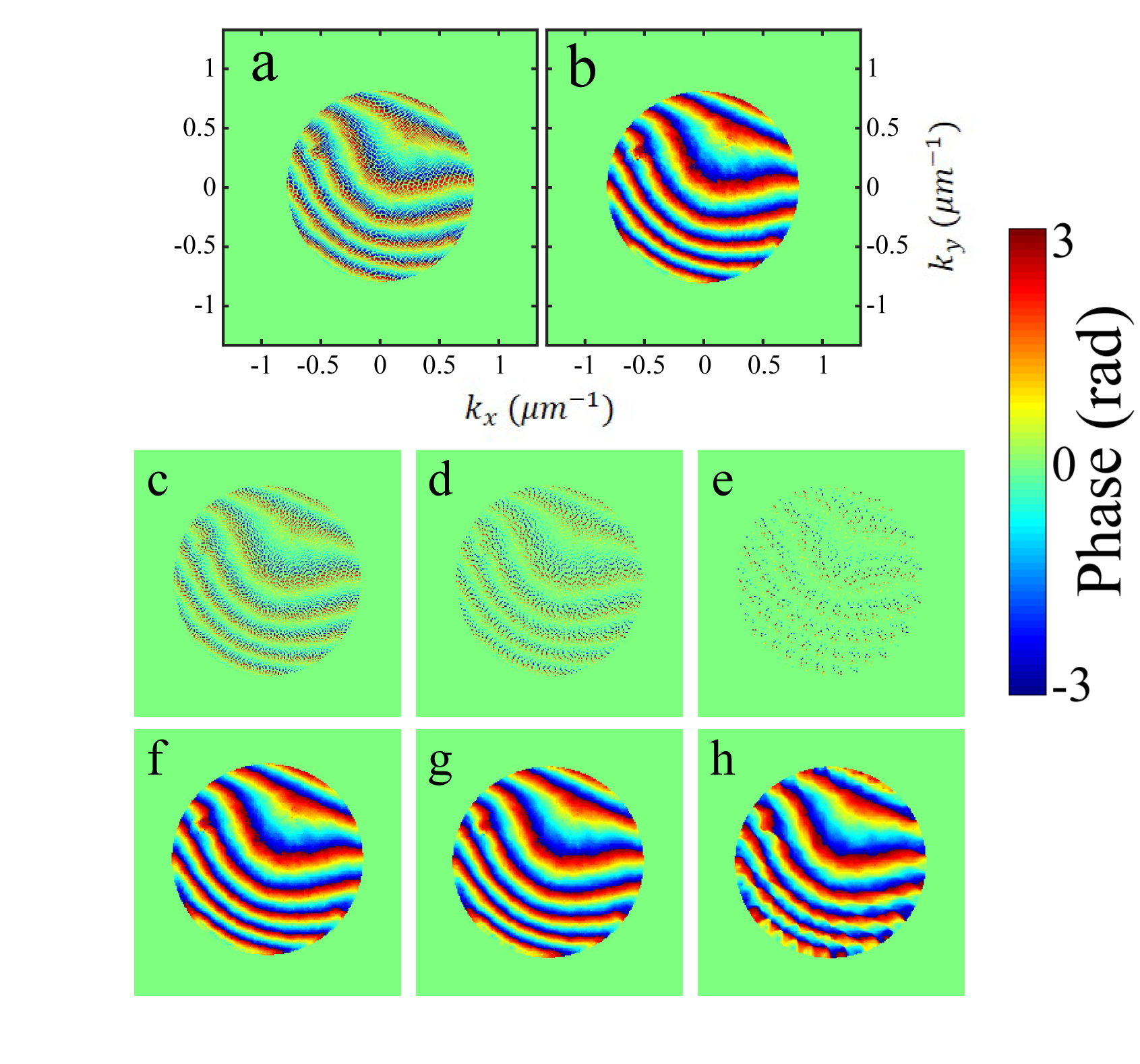


**Supplementary Figure 4.** Construction of a PM by the iterative interpolation process. (a) A PM generated with the full-sized TM by Eq. (9). (b) A PM after the iterative interpolation. (c), (d), and (e) PMs generated by Eq. (9) with reduced sampling densities 50 %, 30 %, and 10% in that order. (f), (g), and (h) PMs after the iterative interpolation processes.

**Supplementary Note 5**

**Assessment of the processing time**

As shown in Fig. 3 in the main text, the result of image reconstruction based on the inverse of the full-sized TM provides the highest imaging quality. This is because that the full-sized TM carries more information about the optical system than PM-LM method does. However, due to the complete characterization of the optical system, the TM inversion method acquires huge amount of data for the TM construction. As a result, the calculation burden for data manipulation and image reconstruction is quite demanding. In our experiment, the reconstruction of all the individual object images by applying the inverse of the full-sized TM took 47.72 seconds and the aperture synthesis for those images took additional 0.50 seconds.

In contrast, PM-LM method does not require the inversion of the minimally sampled TM. The image reconstruction is performed by sequentially applying the PM to the *k*-space and IGT to the position space. The processing time for applying the PM and the IGT took 0.22 second and 0.16 second for each. The image synthesis, which is the common process for both the TM and PM-LM methods, takes 0.50 second. Thus, the PM-LM method took 0.88 seconds in total, which is only 1.8 % compared to the inversion method with the full-sized TM. The comparison of the detailed procedures for image processing is presented in Supplementary Table 1. In these time measurements for image processing, custom-built scripts by Matlab R2015a (MathWorks Inc.) on a standard desktop computer with a 3.60GHz of CPU (i7-6850K, Intel) and 128Gb of RAM were used.

|  |  | $T^{-1}$ | PM-LM |
| --- | --- | --- | --- |
| Pre-calibration  Steps | Measurement of TM | 100.00s | 10.00s |
|  | Construction of TM | 582.83s | 58.81s |
|  | Inversion of TM | 3858.99s | ̶ |
|  | Construction of PM | ̶ | 4.77s |
|  | Iteration for PM (20 cycles) | ̶ | 2.96s |
|  | Construction of LM | ̶ | 33.53s |
|  | Determination of IGT | ̶ | 0.16s |
|  |  | 4541.82s | 110.23s |
| Reconstruction  Steps | Applying $T^{-1}$ | 47.72s | ̶ |
|  | Applying PM | ̶ | 0.22s |
|  | Applying IGT | ̶ | 0.16s |
|  | Synthesizing images | 0.50s | |
|  | | 48.22s | 0.88s |

**Supplementary Table 1.** Comparison of the processing times required for the image corrections with the inverse of the full-sized TM and PM-LM with the minimally sampled TM.

Our PM-LM method is more efficient not only in the reconstruction procedures but also in the pre-calibration steps as shown in Supplementary Table 1. Note that the pre-calibration steps don’t need to be done multiple times unless the optical configuration of the system changes. In the inversion method with the full-sized TM, the TM elements are measured and processed into complex images to form a shape of matrix. And then the inverse of the matrix is calculated for being ready for the image reconstruction. This is the most time-consuming procedure in the inversion method. Thus, the overall taken time for all the pre-calibration steps for inversion method was about 4542 seconds with the same computer.

In the PM-LM method, due to the minimal size of the TM elements, the measurement and construction time for the minimally sampled TM is decreased up to ten-folds. In addition, the major reduction in time came from the inversion-free image processing. Due to the absence of the most-time consuming procedure in the PM-LM method, the overall time for all the pre-calibration steps was about 110 seconds even with additional steps associated with PM and LM constructions, and IGT determination.

**Supplementary Note 6**

**Investigation of the reconstruction performance with various sampling ratio for TM**

In Supplementary Fig. 4, it was shown that the constructed PM distribution remained the same up to 10 % down sampling of the full-sized TM. For more systematic comparison, the USAF target image obtained in Fig. 3 in the main text was reconstructed by both the methods with the same down sampling ratios for the TM from its full size as 10 %, 30 %, 50 %, and 100 %. Supplementary Figure 5 (a) shows the reconstruction results by applying the inverses of the TMs with those different down sampling ratios. With the decrease of the TM elements, the reconstruction quality is monotonically degraded. Even with half the sampling, most of the fine structures disappear and the bigger patterns are also covered by significant noise. As a metric for the reconstruction quality, we calculated the image-to-image correlation between the reconstructed images and the original reference image. As presented in Supplementary Fig. 5(c), the reconstruction quality significantly drops with the lower sampling ratio for the TM construction.

On the other hand, in the PM-LM method, the reconstruction results are almost unaffected as shown in Supplementary Fig. 5(b). The image-to-image correlation also confirmed that the reconstruction performance was maintained constant up to 10 % down sampling of the TM. With the lower sampling ratio than 10 %, the reconstruction quality drops due to the failure of the interpolation for the PM construction. The preservation of the reconstruction performance is attributed to the fact that the phase value in the PM varies so slowly that the interpolation process works up to 10 % down sampling of the TM. If the sampling ratio drops below 10 %, the interpolation starts to fail to fill the missing phase values in the PM construction. Generally, the minimum sampling ratio for the TM, which retains the reconstruction performance, depends on the complexity of the angle-dependent phase retardation of the optical system.


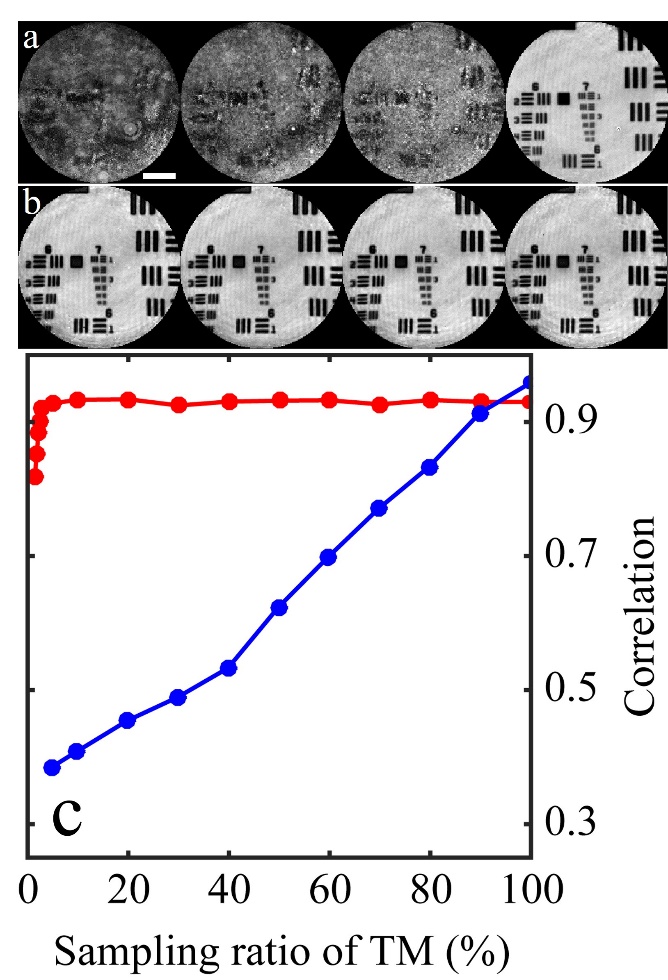


**Supplementary Figure 5.** Image reconstructions by TM and PM-LM methods with different sampling ratios for the TM. (a) USAF target images by the TM inversion with 10, 30, 50, 100% of the TM elements. (b) Same as (a), but by the PM-LM method. (c) Image-to-image correlation between the reconstruction images (blue for TM and red for PM-LM) and the reference image shown in Fig. 3(a). Scale bar: 100 μm.

**Supplementary Note 7**

**Applicability of the method to various aberrations**

In the main text, we have shown that the aberration induced by the FE lens was appropriately corrected by our method and its performance was similar to that by the conventional TM method. Here we demonstrated the applicability of our method to more general cases. In the setup shown in Supplementary Note 2, a molded glass aspheric lens (Thorlabs: C151TMD-A) was used as an objective instead of the FE lens. After the installation, the imaging NA of the aspheric lens was 0.15. As done in Fig. 3 in the main text, 100 images for a USAF resolution target were acquired at different illumination angles up to 0.13 NA for imaging purposes, such as the simple averaging or the aperture synthesis. Thus the overall imaging NA reached 0.28 in total.

We prepared three different cases for the demonstration. As a first, we placed the aspheric lens in the in-focus plane without a tilt from the optical axis as shown in Supplementary Fig. 6(a). As a usual imaging, all the taken intensity images were averaged to produce a bright-field equivalent image. As presented in Supplementary Fig. 6(b), the aberration caused by the aspheric lens was imposed on the object image. Similar to the FE lens, it lost the focus gradually along the radial direction. Particularly, the fine details of the object were significantly blurred out along the edge region. For the spatial distortion, unlike the FE lens, the image shows a barrel distortion, where the magnifications along the horizontal and vertical directions are larger than those along the two diagonal directions. Since the lens was aligned symmetrically, the distortion also occurred almost symmetrically over the field of view.

As done in the main text, we measured the TM, and created the PM and LM for the distortion correction. The PM pattern for the in-focus aspheric lens was shown in Supplementary Fig. 6(c). After sequentially applying the PM and the LM to the distorted image, the final image free from the distortion is presented in Supplementary Fig. 6(d). Now the focal plane became flat over the entire field of view and the barrel distortion was substantially suppressed.

Next, we moved the aspheric lens along the optical axis by about 500 μm to induce a defocusing effect as shown in Supplementary Fig. 6(e). After the defocus, the object image was completely blurred out as shown in Supplementary Fig. 6(f). The PM for the correction of the defocus is presented in Supplementary Fig. 6(g). The characteristic concentric phase distribution associated with the defocus can be seen in the created PM pattern. After applying the PM and LM, all the distortions were appropriately handled. In Supplementary Fig. 6(h), the image was reconstructed with the right focus and no distortion.

Finally, we tilted the aspheric lens by about 30 degrees from the optical axis to induce an asymmetric aberration as presented in Supplementary Fig. 6(i). Then the distortion imposed on the image was asymmetric, i.e., the focal and the spatial distortions were biased along one side of the image, as shown in Supplementary Fig. 6(j). The barrel distortion also resided on the object image. The decrease in the field of view along the horizontal direction was due to the shadow of the aspheric lens mount caused by the rotation. The corresponding PM pattern in Supplementary Fig. 6(k) shows the phase distribution reflecting the asymmetry of the aberration. After applying our method, the corrected image is presented in Supplementary Fig. 6(l). Similar to the previous cases, all the distortions were figured out within the field of view.

Since the PMs contain the information about the aberrations, their distribution represent the distortion characteristics of the corresponding optical systems as shown in Supplementary Figs. 6(c), (g), and (k). As shown in Supplementary Fig. 6(c), the deviation of the phase from the flat pattern is minimal when the aspheric lens was aligned in focus and on axis. But, the complexity of the phase distribution increased due to the addition of the defocusing or the tilting effect. The aberration strengths[^6^](#_ENREF_6),[^7^](#_ENREF_7) were measured 1.56, 1.97, and 2.34 radians for Supplementary Figs. 6(c), (g), and (k), respectively. The increase of the complexity in the PM pattern also affected the minimum sampling ratio of the TM to create the PM. The minimum sampling ratios were 10, 15, and 20 % for each case, which were determined by the success of the iterative interpolation process during the creation of the PMs.


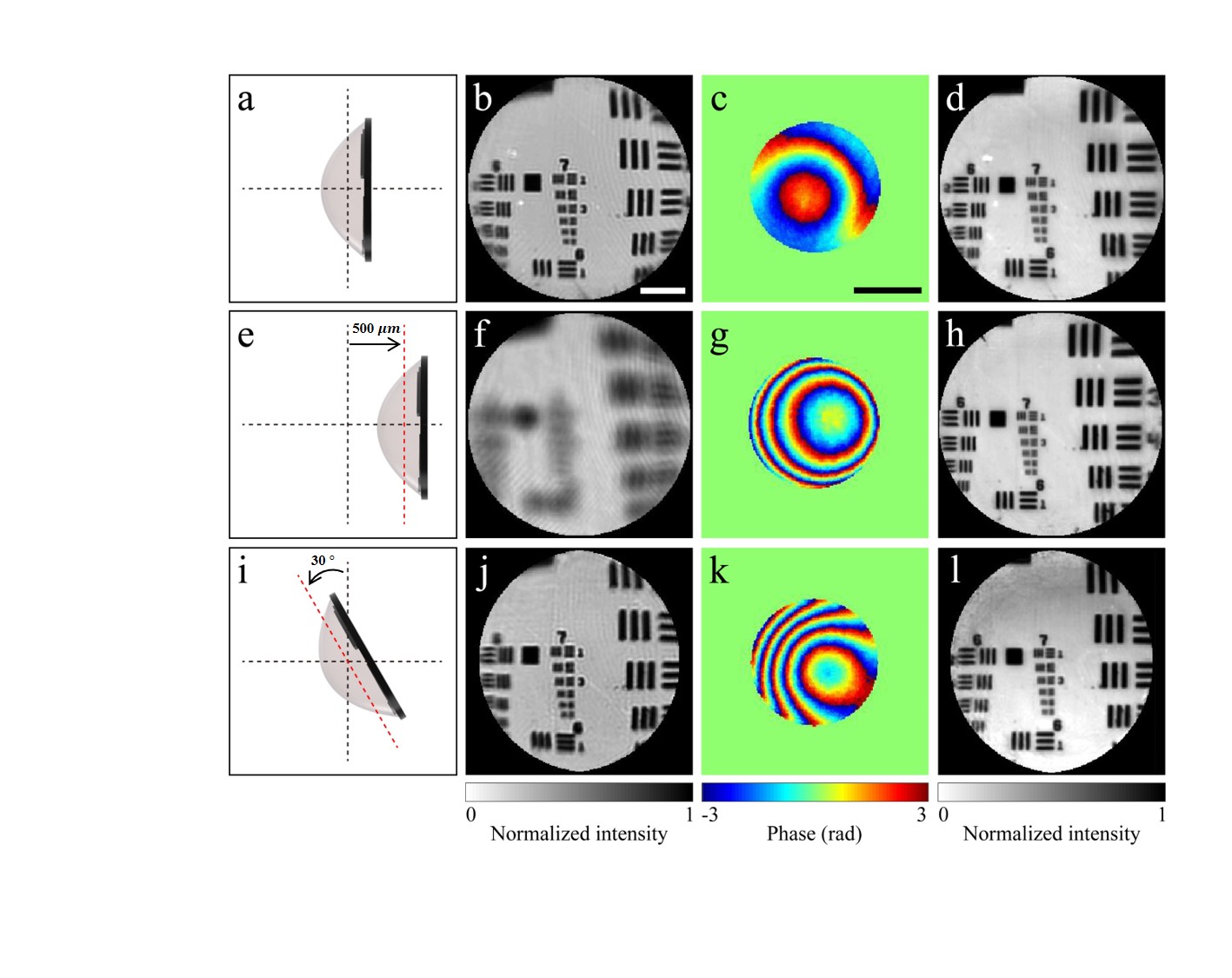


**Supplementary Figure 6.** Image reconstructions by the PM-LM method under various types of aberration. (a) In-focus experiment. The lens was placed in the right focus. (b) The output image. (c) The PM pattern. (d) The corrected image. (e) Defocus experiment. The lens was moved by 500 μm along the axial direction. (f) Output image. (g) The PM pattern. (h) The corrected image. (i) Tilt experiment. The lens was tilted introducing an angle of 30 degrees between the geometrical axis and the optical axis. (j) Output image. (k) The PM pattern. (l) The corrected image. In (b), (d), (f), (h), (j), and (l), scale bar: 100 μm. In (c), (g), and (k), scale bar: 1 μm^-1^.

To evaluate the reconstruction performance, we used the same method as described in the main text, comparing the reconstruction results with those by the conventional TM method using the inversion of the full-sized TM constructed with 10,000 elements. We measured the contrast and SNR of the fine features of the USAF target. The corrected images by the PM-LM method showed contrast (SNR) of 94.2 % (92.0 %), 97.6 % (92.5 %), and 95.5 % (96.6 %) compared to those by the full-sized TM method for Supplementary Figs. 6(d), (h), and (l), respectively. The successful correction of the images with high contrast and SNR means that, our method can be applied to more general situations where the formation of the aberration is more complicated. Even though the degree of aberration varies depending on the optical system, our method can provide a reasonable level of reconstruction performance consistently.

**Supplementary References**

1 Choi, Y. *et al.* Overcoming the Diffraction Limit Using Multiple Light Scattering in a Highly Disordered Medium. *Phys Rev Lett* **107**, doi:ARTN 023902

10.1103/PhysRevLett.107.023902 (2011).

2 Choi, Y., Yoon, C., Kim, M., Choi, W. & Choi, W. Optical Imaging With the Use of a Scattering Lens. *Ieee J Sel Top Quant* **20**, doi:Artn 6800213

10.1109/Jstqe.2013.2275942 (2014).

3 Choi, Y. *et al.* Scanner-Free and Wide-Field Endoscopic Imaging by Using a Single Multimode Optical Fiber. *Phys Rev Lett* **109**, doi:ARTN 203901

10.1103/PhysRevLett.109.203901 (2012).

4 Ikeda, T., Popescu, G., Dasari, R. R. & Feld, M. S. Hilbert phase microscopy for investigating fast dynamics in transparent systems. *Opt Lett* **30**, 1165-1167 (2005).

5 Choi, Y. *et al.* Synthetic aperture microscopy for high resolution imaging through a turbid medium. *Opt Lett* **36**, 4263-4265, doi:Doi 10.1364/Ol.36.004263 (2011).

6 Mahajan, V. N. & Diaz, J. A. Imaging characteristics of Zernike and annular polynomial aberrations. *Appl Optics* **52**, 2062-2074 (2013).

7 Sheppard, C. J. R. Marechal condition and the effect of aberrations on Strehl intensity. *Opt Lett* **39**, 2354-2357 (2014).
